# Testing the Efficacy of ‘Unlearning’, a Mindfulness and Compassion-Based Programme for Cultivating Nonviolence in Teenagers: A Randomised Controlled Trial

**DOI:** 10.3389/fpsyg.2021.717736

**Published:** 2021-12-16

**Authors:** Irene Delgado-Suárez, Yolanda López-del-Hoyo, Javier García-Campayo, Adrián Pérez-Aranda, Marta Modrego-Alarcón, María Beltrán-Ruiz, Santiago Gascón, Jesús Montero-Marín

**Affiliations:** ^1^Institute of Health Research of Aragon (IIS Aragón), Miguel Servet University Hospital, Zaragoza, Spain; ^2^Department of Psychology and Sociology, Faculty of Social and Human Sciences, University of Zaragoza, Zaragoza, Spain; ^3^Primary Care Prevention and Health Promotion Research Network (RedIAPP), Zaragoza, Spain; ^4^Department of Basic, Developmental and Educational Psychology, Autonomous University of Barcelona, Barcelona, Spain; ^5^Department of Psychiatry, Warneford Hospital, University of Oxford, Oxford, United Kingdom

**Keywords:** mindfulness, compassion, nonviolence, randomised controlled trial, teenagers

## Abstract

**Background:** Most programmes developed to reduce aggressive attitudes among teenagers are based on cultivating nonviolence, a construct that has been related to compassion and, more indirectly, mindfulness. This study aims at testing the efficacy of ‘Unlearning’, a mindfulness and compassion-based programme, for reducing aggressive attitudes in adolescents.

**Method:** A sample of 164 students from three high schools in Zaragoza (Spain) participated in the study. They were randomly assigned to (1) ‘Unlearning’, or (2) relaxation programme. Three assessment points were established: baseline, post-treatment and a 4-month follow-up. The outcome variables were the subscales of the ‘Attitudes Toward Social Aggression Scale’. Mindfulness and compassion were assessed as secondary outcomes.

**Results:** ‘Unlearning’ did not produce changes in the primary outcomes, but significant effects were observed post-treatment in self-compassion; and in the follow-up, in self-compassion and mindfulness. The control group did not experience any change post-treatment, but a significant effect in mindfulness was observed in the follow-up. The intergroup analyses indicated that ‘Unlearning’ improved self-compassion, both post-treatment (*t* = −2.48, *p* = 0.014) and after 4-months (*t* = −2.03, *p* = 0.044), although these results were not statistically significant after correcting for multiple comparisons.

**Conclusion:** ‘Unlearning’ did not produce significant reductions in aggressive attitudes compared to the control group. The low baseline levels may have hindered the efficacy of the interventions. ‘Unlearning’ showed potential to improve self-compassion, which is related to nonviolence, and this may have positive implications for the adolescents. Future interventions should include teachers and families to enhance the effectiveness of the programmes.

## Introduction

Teenagers constitute a population in high risk of experiencing different types of violence. Bullying represents the most common expression of violence among adolescents (Modecki et al., 2014; Smith, 2016). Despite data regarding its prevalence has shown variability, high rates of victimisation have generally been reported; in Spain, 9.3% of the students of secondary centres have experienced bullying or cyberbullying (Observatorio de la Convivencia Escolar, 2016). Thus, there is a wide consensus regarding the need of implementing policies addressed at reducing aggressive attitudes among teenagers.

For that purpose, different programmes have been designed, and most of them share some key elements, such as cultivating nonviolence. Nonviolence can be defined by a series of interconnected values and principles: not killing would be the very first, and subsequently, not producing harm or suffering to oneself or other sentient beings (López-Martínez, 2004; Loy, 2018). Many authors have underlined that, contrary to a general belief that considers nonviolence as a passive act, it involves a strong positive exercise of loving other beings (Nagler, 2014; Garcia-Campayo, 2020), for which it has been related to compassion (Wang, 2018), defined as an orientation to be sensitive towards suffering – both own and others’ – and a commitment to relieve it by recognising its universality and the ability to meet that pain with equanimity (Feldman and Kuyken, 2011; Macbeth and Gumley, 2012).

Among the different approaches that have been conducted for promoting nonviolence and other related concepts, some programmes have presented very promising results. For instance, the ‘Olweus Bullying Prevention Program’ focused on aspects, such as discipline, authority, limits and consequences, rewarding pro-social attitudes (Olweus and Limber, 2010), and it achieved significant changes in the ‘school culture’ regarding bullying (Olweus et al., 2020). On the other hand, the ‘KiVa’ programme was addressed at teaching the teachers effective strategies for managing harassment situations and providing teenagers emotional education. This programme also produced significant decreases in self-reported rates of bullying and victimisation (Green et al., 2020). Another example would be the ‘Friendly Schools’ programme, based on promoting assertiveness and emotional regulation, among other abilities; a significant decrease in reported bullying perpetration was observed after the programme (Cross et al., 2019). Despite the variability that the different programmes present, a meta-analysis indicated that these interventions produce significant reductions of around 20% in bullying rates (Ttofi and Farrington, 2011).

As abovementioned, nonviolence has been sometimes identified with the concept of compassion, and some studies have analysed the role of self-compassion and other related constructs, such as mindfulness, on aggressive attitudes and victimisation (Dávila Gómez et al., 2020). Mindfulness is defined as a present-focused, non-judgmental awareness whose practice is associated with a wide range of positive mental health-related outcomes, also in children and teenagers (Zoogman et al., 2015; Carsley et al., 2018). In the case of nonviolence, mindfulness has been considered relevant for its capacity to promote inner peace (Nagler, 2004). In the adult population, some studies have found promising evidence: the Freedom project, focused on training prisoners in nonviolent communication, observed significant effects of a mindfulness and compassion-based intervention in different outcomes, including anger (Suarez et al., 2014). Wang (2018) also used mindfulness along with other techniques to enhance nonviolence to the self in educators. In the case of teenagers, while some studies have not found significant effects of mindfulness and compassion-based interventions on violence-related constructs (Huppert and Johnson, 2010; Schonert-Reichl and Lawlor, 2010; Tharaldsen, 2012; Wongtongkam et al., 2015; Rawlett et al., 2019), some others have reported promising evidence of the effect of such programmes on empathic and pro-environmental attitudes (Jalón et al., 2020) and on constructs such as hostility, lack of social compromise, aggresivity and impuslivity (Nelson-Gray et al., 2006; Franco et al., 2016; Salmoirago-Blotcher et al., 2019; Georgiou et al., 2020). However, these studies have been conducted on specific samples which do not represent the general population of teenagers, and other limitations, such as the lack of an active control condition or a follow-up assessment, imply that these results should be considered preliminary and that further research is needed.

This study aimed at analysing the efficacy of a mindfulness and compassion-based programme, specifically designed for this project, named ‘Unlearning’. The intervention was addressed at cultivating nonviolence through the practice of mindfulness and self-compassion in teenagers who were studying secondary education. ‘Unlearning’ was compared to an active control condition, equivalent in terms of duration, consisting of a programme addressed at teaching relaxation techniques and time management. The hypothesis of the researchers was that ‘Unlearning’, which specifically addressed self-compassion attitudes and mindfulness, would significantly decrease aggressive attitudes among teenagers compared to the control condition, and these effects were expected to be maintained in the 4-month follow-up assessment.

## Materials and Methods

### Participants

Students from three secondary education centres in Zaragoza (Spain) participated in the present study. Once the informed consent had been signed by both the student and their legal guardian, each participant was randomised to one of the following study arms: (1) ‘Unlearning’; or (2) active control group (i.e. relaxation programme). The exclusion criteria for participating in the study were not being able to attend to the sessions due to schedule incompatibilities and/or changing of school during the following year. The sample size was calculated assuming a large effect of ‘Unlearning’ vs. the relaxation programme in the primary outcomes, an equal 1:1 allocation rate and *d* = 0.80 when comparing the two study arms. It was estimated at 146 patients (73 per group); assuming an attrition rate of 10% at follow-up, the total sample size required was established at 162 patients (81 per group).

### Procedure

The present study was conducted during two academic courses: 2018–19 and 2019–20. This project was announced in the 2nd Congress of Mindfulness in Education (2017) and the 5^th^ International Mindfulness Congress (2018), which were held in Zaragoza, and in different mindfulness-based courses. Teachers who showed interest in the project were contacted for an in-depth explanation of the aims and procedures, which were afterwards presented to the school’s head of studies for approval. Three of the initial seven interested secondary education centres were committed to the study requirements (i.e. schedules and students’ randomisation); two of them were public schools and the other one was private. Both the students and their families or legal guardians were offered information about the study *via* an informative meeting and an email answering frequently asked questions, along with the informed consent; those who signed the document were included in the study.

The baseline measures were assessed before randomisation, which was conducted by a member of the research team who was not related to this project using Epidat 4.2. Following the methods used in previous randomised controlled trials implying teenagers (Johnstone et al., 2016), the study conditions remained blind for the participants, although the nature of the intervention was clear after some sessions. Both interventions were delivered in parallel in each high school, and they were conducted by external professionals. After 8 weeks, the post-treatment assessment was conducted, and a 4-month follow-up assessment was also performed; this last evaluation was supposed to be conducted after 6 months, but the restrictions derived from the COVID-19 pandemic in the first semester of 2020 forced the research team to adapt the schedule and, anticipating a possible advance of the end of the school year, the follow-up assessment was conducted in April 2020 *via* SurveyMonkey.

### Interventions

#### ‘Unlearning’

This programme consists of a weekly 45–50 min session for 8 weeks conducted by a health professional with specific formation in mindfulness and compassion-based interventions. The programme includes teachings, daily simple exercises, meditations, visualisations and specific practices to augment the teenagers’ ability to be considerate and kind towards themselves and others and to increase present-focused attention. A more detailed description of the intervention can be found in Delgado Suárez (2020).

‘Unlearning’ was designed by the research group considering Crane et al. (2017) standards for mindfulness-based interventions and pedagogic recommendations for implementing mindfulness in educational contexts, such as offering interactive, experiential, student-based learning (Santorelli, 2000). Regarding the study population, some adaptations were made so the programme was easily followed by teenagers: explicit teaching of abilities, interactive methods based on teenage-related experiences and facilitating resources for implementing mindfulness and compassion in the daily life (Kuyken et al., 2013). In addition, some parts of the programme were synthetised and more attention was granted to identifying the student’s needs through the practice of mindfulness and compassion, with the purpose of overcoming common limitations regarding teenager’s lack of interest or compromise towards mindfulness (McKeering and Hwang, 2019).

#### Relaxation Programme

The active control group presented an equivalent format to ‘Unlearning’ (i.e. 8 sessions of 45–50 min, once per week) with no overlap in terms of content. This programme also included teachings, daily simple exercises, visualisations and specific practices to promote relaxation and increase the student’s time management ability, considered a strong predictor of academic performance (Mercanlioglu, 2010; Aeon et al., 2021). The relaxation exercises were based on Jacobson’s techniques (Schwarz and Schwarz, 2017). A session-by-session description of the intervention can be found elsewhere (Delgado Suárez, 2020).

### Measures

#### Nonviolent Attitudes and Behaviours

The Attitudes Toward Social Aggression Scale (ATSAS; De La Villa Moral, 2005) is a 48-item self-report measure to assess attitudes towards violence and harassment among teenagers. It includes 3 subscales which represent the primary outcomes of the present study: (1) cognitive dimension, consisting of 14 items that assess the students’ perceptions about using violence, both physical and verbal, towards their classmates (e.g. ‘violence is the last resort’); (2) affective dimension, which includes 8 items that examine the degree of identification or rejection that both aggressors and victims generate in the teenager (e.g. ‘I do not want to have violent friends’); and (3) behavioural dimension, which consists of 26 items and refers to behavioural dispositions and previous experiences related to violence (e.g. ‘I avoid taking part in fights’). Each item was answered in a Likert-type scale (1 = ‘Strongly disagree’, 5 = ‘Strongly agree’). The scores of the subscales ranged from 14 to 70 for the cognitive dimension, from 8 to 40 for the affective dimension and from 26 to 130 for the behavioural dimension. Higher scores indicate higher levels of aggressive attitudes. The questionnaire was developed in Spanish and presented good psychometric properties, including high internal consistency (*α* = 0.89; De La Villa Moral, 2005).

#### Mindfulness

The Child and Adolescent Mindfulness Measure (CAMM; Greco et al., 2011) is a 10-item self-report questionnaire that assesses mindfulness in children and teenagers. Each item (e.g. ‘I pay close attention to my thoughts’) is answered using a Likert scale (0 = ‘Never true’; 4 = ‘Always true’); the total score ranges from 0 to 40, with higher scores reflecting higher levels of mindfulness. The Spanish version of the CAMM has presented good psychometric properties, including adequate internal consistency (*α* = 0.82; Turanzas, 2013).

#### Self-Compassion

The Self-Compassion Scale-Short Form (SCS-SF; Raes et al., 2011) is a 12-item on five-point Likert scale (0 = ‘Almost never’ to 5 = ‘Almost always’) to record how often the respondent behaves kindly and caringly towards themself in difficult life situations (e.g. ‘I try to be loving towards myself when I’m feeling emotional pain’). This questionnaire is an adaptation from the original SCS (Neff, 2003). The present study only used the global SCS-SF score, which is calculated by taking the average score of the 12 items; it ranges from 0 to 5, with higher scores reflecting higher levels of self-compassion. The Spanish version of the SCS-SF has shown high internal consistency (*α* = 0.86; Garcia-Campayo et al., 2014).

### Data Analysis

Sociodemographic data were described at baseline by means of frequencies or means. A visual inspection and the corresponding Chi-squared or Student’s t tests were conducted to ascertain the equal distribution of sociodemographics among groups at baseline.

The primary analysis consisted of the evaluation of the efficacy of ‘Unlearning’ compared to the active control condition for promoting nonviolence; the primary outcomes were the ATSAS’ subscales, which were taken as continuous outcomes. A repeated measures design was performed with an intention to treat basis using multilevel mixed effects linear regression models, including time as an independent variable and subjects and the group of delivery (subgroup) as random effects, focussing our interest at the individual (student) level. Restricted maximum likelihood estimation was used, which produces unbiased estimates in case of small or unbalanced sample sizes (Egbewale et al., 2014). Non-standardised slopes and 95% confidence intervals (CI) were calculated. Effect sizes were assessed using Cohen’s d statistic, calculated by the combined standard deviation in the baseline, weighing the differences between the corresponding marginal means (Morris, 2008). Effect sizes are usually defined as small when *d* = 0.2; medium when *d* = 0.5; and large when *d* = 0.8 (Cohen, 1988). The efficacy of the interventions regarding the SDQ, the CAMM and the SCS-SF was calculated following the same analytical strategy used for the primary analysis. Within-group analyses were also performed for both primary and secondary outcomes.

An alpha level of 0.05 was set, using a two-tailed test. We applied the Benjamini-Hochberg correction for multiple comparisons (Glickman et al., 2014). Data analyses were computed using IBM SPSS v26.0 statistical software.

## Results

### Participant Flow and Compliance

**Figure 1:**
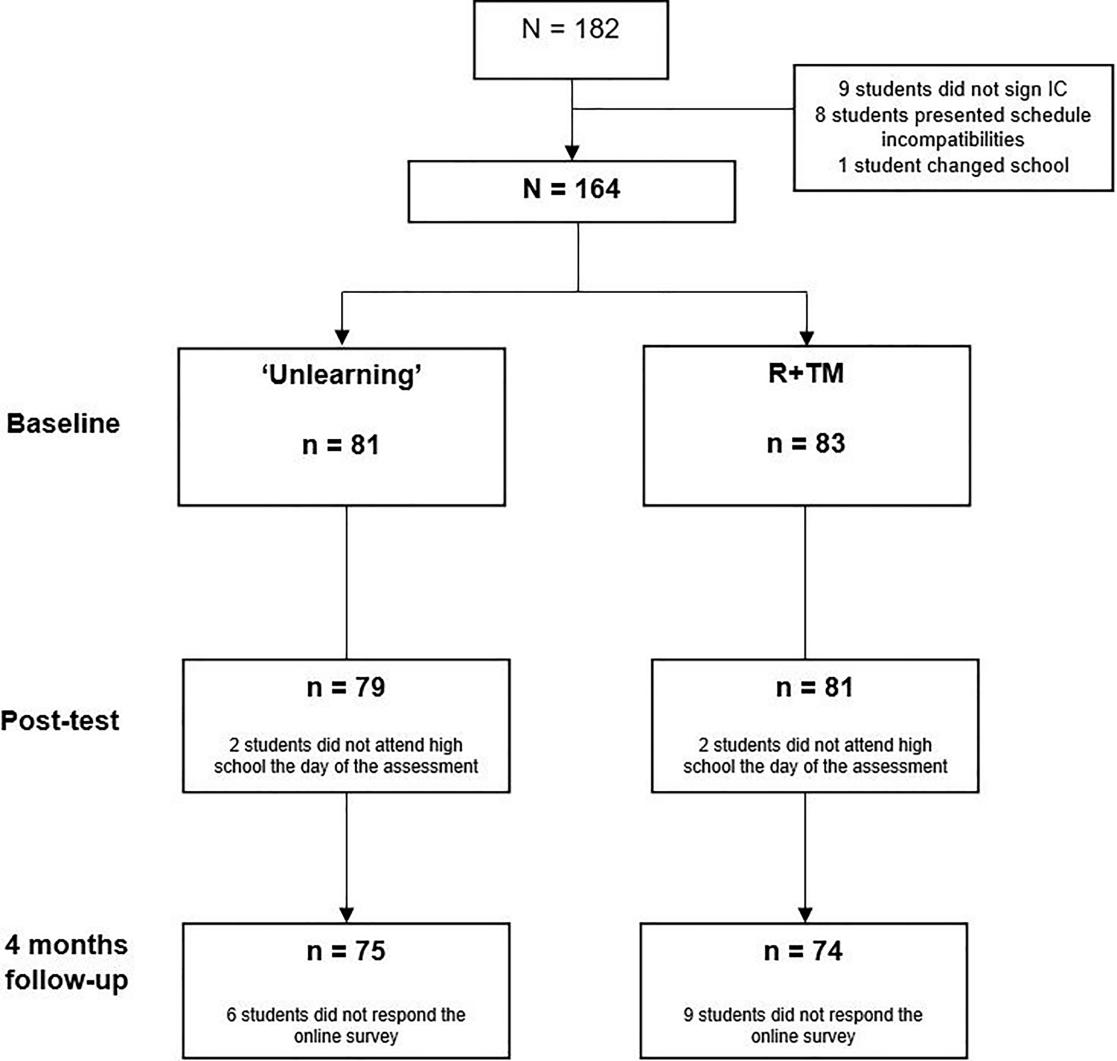
Flow chart of participants in the randomised controlled trial.

A total of 182 students from three secondary education centres conformed the initial sample, although some of them did not meet the inclusion criteria (see Figure 1). Therefore, the final sample consisted of 164 high school students which were randomised to ‘Unlearning’ (*n* = 81) or relaxation (*n* = 83). Most of the participants attended all the sessions in both programmes (83.5%). In the ‘Unlearning’ group, 79 (97.5%) participants provided post-test data, and 75 (92.6%) students performed the 4-month follow-up assessment. For the active control group, the retention rates were 97.6% post-test and 89.2% at follow-up, which did not represent significant differences between the groups.

### Baseline Characteristics of the Sample

The sociodemographic characteristics of the sample are presented in Table 1. No significant differences were appreciated between the ‘Unlearning’ group and the control group in any of the variables. Regarding the baseline levels of the study variables, no significant differences were observed between the groups. A table of correlations has been included as Supplementary Material.

**Table 1:** Sociodemographic characteristics of the sample and baseline levels of nonviolence, mindfulness and compassion.

	Unlearning	Relaxation	*t*/*χ*^2^ (*p*)
	(*n* = 81)	(*n* = 83)	
**Sociodemographic characteristics**			
**Sex, *n* (%)**			
Female	44 (54.3%)	47 (56.6%)	1.97 (0.211)
Male	37 (45.7%)	36 (43.4%)	
**Age, mean (*SD*)**	13.78 (1.09)	13.78 (1.10)	0.03 (0.975)
**School, *n* (%)**			
Public	64 (79%)	64 (77.1%)	0.09 (0.851)
Private	17 (21%)	19 (22.9%)	
**Year of studies, *n* (%)**			
2nd	40 (49.4%)	40 (48.2%)	0.09 (0.958)
3rd	17 (21%)	19 (22.9%)	
4th	24 (29.6%)	24 (29.9%)	
**Study variables**			
**ATSAS, *M* (*SD*)**			
Cognitive	32.74 (7.83)	32.56 (7.56)	0.98 (0.328)
Affective	29.78 (5.24)	30.22 (5.38)	−0.53 (0.597)
Behaviour	56.11 (12.87)	54.47 (14.10)	0.78 (0.438)
**CAMM, *M* (*SD*)**	13.90 (6.78)	14.25 (6.15)	−0.34 (0.735)
**SCS-SF, *M* (*SD*)**	2.83 (0.85)	2.91 (0.95)	−0.53 (0.601)

ATSAS = Attitudes Toward Social Aggression Scale, CAMM = Child and Adolescent Mindfulness Measure, SCS = Self-compassion scale.

### Effects on the Primary Outcomes

The within-group analyses (Table 2) indicated that ‘Unlearning’ did not produce any significant effect on the primary outcomes post-test nor in 4-month follow-up. The relaxation programme showed a significant intragroup effect in the ATSAS ‘Cognitive’ subscale, as a significant increase in the score was observed in the follow-up (*t* = 2.34, *p* = 0.021), although the effect size was small (*d* = 0.25), and this result was not significant after applying the Benjamini-Hochberg correction. The other ATSAS subscales did not present significant effects. The mixed effects model indicated that both interventions were equivalent regarding the primary outcome in every time point, although a tendency close to statistical significance was observed in the ‘Behavioural’ subscale in favour of ‘Unlearning’ (*t* = 1.81, *p* = 0.073, *d* = 0.61; Table 3).

**Table 2:** Raw scores and intragroup differences per group.

	Baseline *M* (*SD*)	Post-test *M* (*SD*)	Follow-up *M* (*SD*)	Wald^a^ *χ*^2^*_df_* (*p*)	LR test^b^ *χ*^2^*_df_* (*p*)	Baseline vs. Post-test			Baseline vs. Follow-up		
						*d*	*B* (95% CI)	*t* (*p*)	*d*	*B* (95% CI)	*t* (*p*)
**‘Unlearning’**											
ASTAS Cognitive	31.56 (7.56)	32.10 (8.70)	31.17 (8.57)	1.0 _(2)_ (0.063)	105.7 _(2)_ (<0.001)	0.07	0.58 (−0.69 to 1.85)	0.85 (0.394)	0.00	0.05 (−1.28 to 1.38)	−0.02 (0.986)
ATSAS Affective	30.22 (5.38)	29.76 (5.76)	29.95 (5.54)	1.1 _(2)_ (0.574)	128.0 _(2)_ (<0.001)	0.09	−0.46 (−1.37 to 0.45)	−0.98 (0.327)	0.07	37 (−1.31 to 0.56)	−0.76 (0.447)
ATSAS Behaviour	54.47 (14.10)	54.97 (18.14)	52.44 (16.06)	2.0 _(2)_ (0.371)	140.5 _(2)_ (<0.001)	0.05	0.43 (−2.09 to 2.95)	0.35 (0.726)	0.17	−1.38 (−3.99 to 1.22)	−1.12 (0.265)
CAMM	14.25 (6.15)	14.44 (5.87)	19.99 (6.93)	61.4 _(2)_ (<0.001)	26.6 _(2)_ (<0.001)	0.03	0.18 (−1.42 to 1.77)	0.23 (0.819)	**0.85**	5.84 (4.20 to 7.48)	**7.08 (<0.001)**
SCS-SF	2.91 (0.95)	3.24 (0.89)	3.25 (0.91)	16.6 _(2)_ (<0.001)	62.0 _(2)_ (<0.001)	**0.38**	0.34 (0.15 to 0.54)	**3.68 (<0.001)**	**0.40**	0.36 (0.16 to 0.56)	**3.65 (<0.001)**
**Relaxation**											
ASTAS Cognitive	32.73 (7.83)	33.78 (7.76)	34.36 (8.71)	5.5 _(2)_ (0.617)	140.4 _(2)_ (<0.001)	0.14	0.91 (−0.50 to 2.33)	1.36 (0.176)	**0.25**	1.76 (0.29 to 3.23)	**2.34 (0.021)** *
ATSAS Affective	29.78 (5.24)	28.96 (5.33)	28.97 (5.31)	4.4 _(2)_ (0.110)	102.5 _(2)_ (<0.001)	0.15	−0.84 (−1.76 to 0.09)	−1.81 (0.074)	0.17	−0.91 (−1.88 to 0.07)	−1.85 (0.067)
ATSAS Behaviour	56.11 (12.87)	57.90 (14.25)	57.32 (14.52)	3.1 _(2)_ (0.216)	140.8 _(2)_ (<0.001)	0.19	1.57 (−0.55 to 3.68)	1.33 (0.185)	0.23	1.77 (−0.48 to 4.01)	1.44 (0.153)
CAMM	13.90 (6.78)	15.30 (6.55)	18.92 (9.25)	36.2 _(2)_ (<0.001)	65.5 _(2)_ (<0.001)	0.23	1.58 (−0.04 to 3.19)	1.88 (0.062)	**0.73**	5.05 (3.38 to 6.72)	**6.09 (<0.001)**
SCS-SF	2.83 (0.85)	2.84 (0.87)	2.88 (0.93)	0.8 _(2)_ (0.700)	74.5 _(2)_ (<0.001)	0.02	0.02 (−0.16 to 0.19)	0.19 (0.847)	0.08	0.08 (−0.10 to 0.26)	0.78 (0.438)

* statistically not significant after applying the Benjamini-Hochberg correction;

Significant effects are presented in bold. ATSAS = Attitudes Toward Social Aggression Scale, CAMM = Child and Adolescent Mindfulness Measure, SCS = Self-compassion scale. *M* = mean. *SD* = standard deviation. ^a^Omnibus test. ^b^Model fit of the mixed model. *d* = effect size. *B* (95% CI) = regression coefficient (95% confidence interval). *t*(*p*) = *t* test (value of *p*).

**Table 3:** Between-group analyses for primary and secondary outcomes (ITT approach).

Outcomes	Unlearning *M* (*SD*)	Relaxation *M* (*SD*)	Wald^a^ *χ*^2^*_df_* (*p*)	LR test^b^ *χ*^2^*_df_* (*p*)	Unlearning vs. Relaxation		
					*d*	*B* (95% CI)	*t*(*p*)
**Primary**							
ATSAS-Cognitive (14–70)			9.6 _(5)_ (0.087)	244.6 _(2)_ (<0.001)			
Baseline	31.56 (7.56)	32.73 (7.83)					
Post-treatment	32.10 (8.70)	33.78 (7.76)			0.19	0.33 (−1.54 to 2.19)	0.34 (0.729)
Follow-up	31.17 (8.57)	34.36 (8.71)			0.37	1.76 (−0.34 to 3.87)	1.65 (0.101)
ATSAS Affective (8–40)			6.6 _(5)_ (0.250)	233.3 _(2)_ (<0.001)			
Baseline	30.22 (5.38)	29.78 (5.24)					
Post-treatment	29.76 (5.76)	28.96 (5.33)			0.15	−0.38 (−1.68 to 0.92)	−0.57 (0.566)
Follow-up	29.95 (5.54)	28.97 (5.31)			0.19	−0.54 (−1.91 to 0.83)	−0.78 (0.439)
ATSAS Behaviour (26–130)			7.1 _(5)_ (0.217)	284.2 _(2)_ (<0.001)			
Baseline	54.47 (14.10)	56.11 (12.87)					
Post-treatment	54.97 (18.14)	57.90 (14.25)			0.34	1.15 (−2.16 to 4.46)	0.68 (0.494)
Follow-up	52.44 (16.06)	57.32 (14.52)			0.61	3.15 (−0.29 to 6.60)	1.81 (0.073)
**Secondary**							
CAMM (0–40)			98.2 _(5)_ (<0.001)	93.3 _(2)_ (<0.001)			
Baseline	14.25 (6.15)	13.90 (6.78)					
Post-treatment	14.44 (5.87)	15.30 (6.55)			0.15	1.36 (−0.95 to 3.67)	1.16 (0.247)
Follow-up	19.99 (6.93)	18.92 (9.25)			0.17	−0.85 (−3.16 to 1.47)	−0.72 (0.471)
SCS-SF (0–5)			24.8 _(5)_ (<0.001)	135.5 _(2)_ (<0.001)			
Baseline	2.91 (0.95)	2.83 (0.85)					
Post-treatment	3.24 (0.89)	2.84 (0.87)			**0.45**	−0.33 (−0.59 to −0.07)	**−2.48 (0.014)** *
Follow-up	3.25 (0.91)	2.88 (0.93)			**0.41**	−0.28 (−0.56 to −0.01)	**−2.03 (0.044)** *

* statistically not significant after applying the Benjamini-Hochberg correction;

Significant effects are presented in bold. ATSAS = Attitudes Toward Social Aggression Scale, CAMM = Child and Adolescent Mindfulness Measure, SCS = Self-compassion scale. *M* = mean. *SD* = standard deviation. ^a^Omnibus test. ^b^Model fit of the mixed model. *d* = effect size. *B* (95% CI) = regression coefficient (95% confidence interval). *t*(*p*) = *t* test (value of *p*).

### Effects on the Secondary Outcomes

The within-group analyses (Table 2) indicated that ‘Unlearning’ produced a significant effect post-test in self-compassion (*t* = 3.68, *p* < 0.001) of small effect size (*d* = 0.38). In the follow-up assessment, that effect was maintained (*t* = 3.65, *p* < 0.001, *d* = 0.40), and a significant increase in mindfulness was observed (*t* = 7.08, *p* < 0.001), which presented a large effect size (*d* = 0.85). The relaxation programme did not produce any significant effect post-test, but only a significant increase in mindfulness in the follow-up (*t* = 6.09, *p* < 0.001) with a moderately large effect size (*d* = 0.73).

The intergroup analyses (Table 3) reflected significant differences between the two study arms in self-compassion both post-test (*t* = −2.48, *p* = 0.014, *d* = 0.45) and in the 4-month follow-up assessment (*t* = −2.03, *p* = 0.044, *d* = 0.41), but both effects lost statistical significance after applying the Benjamini-Hochberg correction.

## Discussion

The results of the present study indicate that the sample presented relatively low baseline levels of aggressive attitudes in the cognitive and the behavioural dimensions (De La Villa Moral, 2005); that would imply that teenagers tended to believe things such ‘violence is the last resort’ or ‘those who are violent end up being rejected by others’ and that they usually acted in a non-impulsive, peaceful way. These low baseline levels could have been in part responsible of the lack of effect of ‘Unlearning’ in these dimensions. Regarding the active control group, a statistically significant increase in the cognitive dimension of social aggression was observed in the follow-up, although the effect size indicated that it was small, and it was not significant after applying the Benjamini-Hochberg correction; thus, this should be studied in future studies before considering it a relevant result. On the other hand, the affective dimension presented relatively high baseline levels of social aggression, reflecting that the students presented tendencies to feel admiration towards those who know how to insult or to enjoy violent movies. This had already been reported by De La Villa Moral (2005), who found that insulting or threatening could be perceived by adolescents as a form of asserting social status in a group. No significant effects were observed in this dimension after the interventions, post-treatment nor in the 4-month follow-up assessment.

This is not the first study to observe lack of effects of mindfulness-based interventions on aggressive attitudes; for instance, Wongtongkam et al. (2015) found that their mindfulness programme was not superior to an inactive control group for reducing violent behaviours among university students. On the other hand, some studies which focused on specific samples, such as teenagers with severe behaviour problems (Franco et al., 2016), oppositional defiant disorder (Nelson-Gray et al., 2006) or hyperactivity (Singh, 2016), found positive results for the mindfulness programmes. In these cases, the interventions were efficacious compared with an inactive control group, but the methodological shortcomings hinder the generalisation of these findings. In view of these results, it could be possible that mindfulness-based interventions could be more efficacious for reducing negative attitudes than for enhancing positive ones, at least in what refers to personal beliefs and behaviours.

Another aspect that could explain the lack of effect of ‘Unlearning’ could be its format. Salmoirago-Blotcher et al. (2019) implemented a mindfulness-based programme which lasted longer and included more sessions per week and found that the programme was more efficacious than an intervention addressed to enhancing resilience and self-esteem, among others; however, the effects were not maintained in a 6-month follow-up assessment, which reflects that the efficacy of the intervention was limited to the short term. This leads to wonder if mindfulness-based programmes are the best approach for reducing aggressive attitudes among adolescents; although there are some methodological shortcomings, there is a significant number of studies indicating that different programmes (not mindfulness-related) produce significant reductions in bullying and victimisation rates (Ttofi and Farrington, 2011). However, it has been observed that the efficacy of anti-bullying programmes decreases notably in teenagers older than 13 (Villanueva Badenes et al., 2013; Yeager et al., 2015), and in some cases, adverse effects (i.e. increasing aggressive attitudes) have been reported after the interventions (Cerezo Ramírez and Sánchez Lacasa, 2013; Campbell et al., 2019). All in all, promoting nonviolence among teenagers constitutes a difficult challenge for educators. Including the students’ families, the teachers and the community could be an aspect of higher relevance for a programme to be effective and finding ways to act not only in class but also in the teenager’s home is also needed.

For what concerns to the secondary outcomes, the intragroup analysis suggests that ‘Unlearning’ significantly promoted mindfulness, although only in the follow-up assessment, but with a large effect size. The active control condition also produced significant improvements in mindfulness in the follow-up, with a moderate effect size. Thus, the intergroup analyses indicated that ‘Unlearning’ did not produce significant effects compared to the relaxation programme in mindfulness, which suggests that both interventions promote improvements in mindfulness skills or that maybe these changes are due to maturation effects related to the passage of time. Previous studies have already found that mindfulness programmes do not improve mindfulness in teenagers more than active control conditions (Huppert and Johnson, 2010; Schonert-Reichl and Lawlor, 2010; Tharaldsen, 2012; Rawlett et al., 2019). Some of these authors have suggested that adolescents may not have developed yet the abilities to successfully integrate and apply mindfulness skills that have been trained in relatively short periods of time. Moreover, mindfulness programmes could lead to experience frustration among teenagers, as they encourage them to be aware of negative common thoughts and feelings instead of avoiding them. Interestingly, meta-analytic results suggest that mindfulness-based interventions seem to be more effective for teenagers with clinical conditions than for the general population (Zenner et al., 2014; Zoogman et al., 2015; Dunning et al., 2019), which could be reflecting that, as abovementioned, these programmes could be more efficacious for reducing negative tendencies in targeted sub-groups rather than improving positive ones, as some authors have suggested (Quach et al., 2016).

On the other hand, ‘Unlearning’ seemed to produce a significant improvement in self-compassion compared to the relaxation programme, both post-treatment and after 4 months, although these effects were not significant after applying the correction for multiple comparisons. Improving self-compassion could be a very positive outcome since it could have major implications for the teenagers, not only in their aggressive attitudes, but also on very common problems they face, such as self-judgment, mood changes and loneliness feelings (Bluth et al., 2016b; Pullmer et al., 2019). A compassion-based programme named ‘Making friends with yourself’ produced significant improvements compared with an inactive control group in self-compassion in a sample of 34 teenagers (Bluth et al., 2016a), and the authors observed a potential buffering effect of self-compassion in protecting adolescents from social stressors (Bluth et al., 2016b). However, in line with our results, the meta-analysis conducted by Wilson et al. (2019) found that compassion-based programmes had significant effects on self-compassion, although not when compared to active control conditions.

Some limitations of the present study need to be acknowledged. First, the sample was formed by teenagers from one city (Zaragoza, Spain), and the high schools were not randomly selected, which limits the external validity of our results. Also, no students of the 1^st^ year of secondary education were included due to schedule incompatibilities. An important aspect to be considered is the use of self-reported measures, which imply a certain bias, and the lack of a more objective measure of aggressive behaviour (e.g. classroom conflict log and expulsions for misconduct); the effect of social desirability in the answers of the ATSAS could be a possible reason behind the low baseline levels of aggressive attitudes in our sample. In this regard, the ATSAS, despite being a validated instrument, could have been complemented with other measures for a better characterisation of the violent/nonviolent attitude (Olweus, 1996; Cajigas et al., 2004). In the same line, mindfulness, which is generally considered a multifaceted construct, could have been assessed using more complete and extensive measures. Finally, regarding the interventions that were applied in the present study, there was no information in relation to home practice, which is a key element for mindfulness and compassion-based programmes to be effective.

## Conclusion

This study found that the mindfulness and compassion-based programme ‘Unlearning’ was not effective for reducing aggressive attitudes in teenagers. Some dimensions of these attitudes already presented low baseline levels, which probably hindered the efficacy of the interventions. ‘Unlearning’ improved self-compassion, although not in a significant manner compared to the active control group; focusing on this outcome may contribute to help adolescents in dealing with some of the most common emotional problems they have to face. The studies conducted on this topic agree on the complexity of measuring nonviolence and the necessity of including teachers and families in programmes addressed at promoting it. Moreover, the lack of effect of ‘Unlearning’ could go in line with what previous authors have suggested regarding mindfulness possibly not being the best approach for addressing these issues among adolescents.

## Data Availability Statement

The raw data supporting the conclusions of this article will be made available by the authors, without undue reservation.

## Ethics Statement

The studies involving human participants were reviewed and approved by Comité de Ética de la Investigación de la Comunidad Autónoma de Aragón (CEICA): PI18/299. Written informed consent to participate in this study was provided by the participants’ legal guardian/next of kin.

## Author Contributions

ID-S: substantial contributions to the conception and design of the work, data acquisition, data analysis, and interpretation of results. YL-d-H and JG-C: substantial contributions to the conception and design of the work and contributions to revising the work critically for important intellectual content. AP-A and JM-M: substantial contributions to data analysis and contributions to drafting and revising the work critically for important intellectual content. MM-A, MB-R, and SG: contributions to drafting and revising the work critically for important intellectual content. All authors agreed to be accountable for the content of the work.

## Funding

ID-S had a FPU predoctoral contract awarded by the Spanish Ministry of Education, Culture and Sports to the Institute of Health Research of Aragon (IIS) in Zaragoza, Spain (FPU16/03565). AP-A has a ‘Sara Borrell’ research contract from the Instituto de Salud Carlos III (CD20/00181). This research was funded in whole, or in part, by the Welcome Trust (JM-M; 104908/Z/14/Z). This research is funded by a FIS grant from the Carlos III Health Institute of the Ministry of Spain Economy and Competitiveness (PI19 / 00805), DGA group (B17-20R) and the Network for Prevention and Health Promotion in Primary Care (RD16 / 0007 / 0005) grant from the Carlos III Health Institute of the Ministry of Economy and Competitiveness co-financed with ERDF funds from the European Union.

## Conflict of Interest

The authors declare that the research was conducted in the absence of any commercial or financial relationships that could be construed as a potential conflict of interest.

## Publisher’s Note

All claims expressed in this article are solely those of the authors and do not necessarily represent those of their affiliated organizations, or those of the publisher, the editors and the reviewers. Any product that may be evaluated in this article, or claim that may be made by its manufacturer, is not guaranteed or endorsed by the publisher.

## Supplementary Material

The Supplementary Material for this article can be found online at: https://www.frontiersin.org/articles/10.3389/fpsyg.2021. 717736/full#supplementary-material

## References

[ref1] Aeon B. Faber A. Panaccio A. (2021). Does time management work? A meta-analysis. PLoS One 16:e0245066. doi: 10.1371/journal.pone.0245066, PMID: 33428644 PMC7799745

[ref2] Bluth K. Gaylord S. A. Campo R. A. Mullarkey M. C. Hobbs L. (2016a). Making friends with yourself: A mixed methods pilot study of a mindful self-compassion program for adolescents. Mindfulness 7, 479–492. doi: 10.1007/s12671-015-0476-6, PMID: 27110301 PMC4838201

[ref3] Bluth K. Roberson P. N. E. Gaylord S. A. Faurot K. R. Grewen K. M. Arzon S.. (2016b). Does self-compassion protect adolescents from stress? J. Child Fam. Stud. 25, 1098–1109. doi: 10.1007/s10826-015-0307-3, PMID: 26997856 PMC4793986

[ref37] Cajigas N. Kahan E. Luzardo M. Najson S. Zamalvide G. (2004). Escala de agresión entre pres para adolescentes y principales resultados. Acción Psicológica 3, 173–186. doi: 10.5944/ap.3.3.511

[ref4] Campbell A. J. Lanthier R. P. Weiss B. A. Shaine M. D. (2019). The impact of a Schoolwide mindfulness program on adolescent well-being, stress, and emotion regulation: A nonrandomized controlled study in a naturalistic setting. J. Child Adolesc. Couns. 5, 18–34. doi: 10.1080/23727810.2018.1556989

[ref5] Carsley D. Khoury B. Heath N. L. (2018). Effectiveness of mindfulness interventions for mental health in schools: a comprehensive meta-analysis. Mindfulness 9, 693–707. doi: 10.1007/s12671-017-0839-2

[ref6] Cerezo Ramírez F. Sánchez Lacasa C. (2013). Eficacia del programa CIP para la mejora de la convivencia escolar y la prevención del bullying en alumnos de Educación Primaria. Apunt. Psicol. 31, 173–181.

[ref7] Cohen J. (1988). Statistical Power Analysis for the Behavioral Sciences. Hillsdale, NJ: Erlbaum Psych Press.

[ref8] Crane R. S. Brewer J. Feldman C. Kabat-Zinn J. Santorelli S. Williams J. M. G.. (2017). What defines mindfulness-based programs? The warp and the weft. Psychol. Med. 47, 990–999. doi: 10.1017/S0033291716003317, PMID: 28031068

[ref9] Cross D. Runions K. C. Shaw T. Wong J. W. Y. Campbell M. Pearce N.. (2019). Friendly schools universal bullying prevention intervention: effectiveness with secondary school students. Int. J. Bull. Prev. 1, 45–57. doi: 10.1007/s42380-018-0004-z

[ref10] Dávila Gómez M. Dávila Pino J. Dávila Pino R. (2020). Self-compassion and predictors of criminal conduct in adolescent offenders. J. Aggress. Maltreat. Trauma 29, 1020–1033. doi: 10.1080/10926771.2019.1697778

[ref11] De La Villa Moral M. (2005). Actitudes socioconstruidas ante la violencia bullying en estudiantes de secundaria. Anu. Psicol. 36, 61–81. doi: 10.1344/anuario.any.volum.numero

[ref12] Delgado Suárez I. (2020). “Desaprendiendo” Programa de mindfulness y compasión para el cultivo de la noviolencia en Educación Secundaria Obligatoria: un estudio controlado aleatorizado/Irene Delgado Suárez. Available at: http://zaguan.unizar.es (Accessed May 4, 2021).

[ref13] Dunning D. L. Griffiths K. Kuyken W. Crane C. Foulkes L. Parker J.. (2019). Research review: The effects of mindfulness-based interventions on cognition and mental health in children and adolescents – a meta-analysis of randomized controlled trials. J. Child Psychol. Psychiatry Allied Discip. 60, 244–258. doi: 10.1111/jcpp.12980, PMID: 30345511 PMC6546608

[ref14] Egbewale B. E. Lewis M. Sim J. (2014). Bias, precision and statistical power of analysis of covariance in the analysis of randomized trials with baseline imbalance: A simulation study. BMC Med. Res. Methodol. 14:49. doi: 10.1186/1471-2288-14-49, PMID: 24712304 PMC3986434

[ref15] Feldman C. Kuyken W. (2011). Compassion in the landscape of suffering. Contemp. Budd. 12, 143–155. doi: 10.1080/14639947.2011.564831

[ref16] Franco C. Amutio A. López-González L. Oriol X. Martínez-Taboada C. (2016). Effect of a mindfulness training program on the impulsivity and aggression levels of adolescents with behavioral problems in the classroom. Front. Psychol. 7:1385. doi: 10.3389/fpsyg.2016.01385, PMID: 27713709 PMC5031764

[ref17] Garcia-Campayo J. (2020). La práctica de la compasión. Amabilidad con los demás y con uno mismo. Madrid: Siglantana.

[ref18] Garcia-Campayo J. Navarro-Gil M. Andrés E. Montero-Marin J. López-Artal L. Demarzo M. M. P. (2014). Validation of the Spanish versions of the long (26 items) and short (12 items) forms of the self-compassion scale (SCS). Health Qual. Life Outcomes 12:4. doi: 10.1186/1477-7525-12-4, PMID: 24410742 PMC3896764

[ref19] Georgiou S. N. Charalambous K. Stavrinides P. (2020). Mindfulness, impulsivity, and moral disengagement as parameters of bullying and victimization at school. Aggress. Behav. 46, 107–115. doi: 10.1002/ab.21876, PMID: 31736085

[ref20] Glickman M. E. Rao S. R. Schultz M. R. (2014). False discovery rate control is a recommended alternative to Bonferroni-type adjustments in health studies. J. Clin. Epidemiol. 67, 850–857. doi: 10.1016/j.jclinepi.2014.03.012, PMID: 24831050

[ref21] Greco L. A. Baer R. A. Smith G. T. (2011). Assessing mindfulness in children and adolescents: development and validation of the child and adolescent mindfulness measure (CAMM). Psychol. Assess. 23, 606–614. doi: 10.1037/a0022819, PMID: 21480722

[ref22] Green V. A. Woods L. Wegerhoff D. Harcourt S. Tannahill S. (2020). An evaluation of the KiVa anti-bullying program in New Zealand. Int. J. Bull. Prev. 2, 225–237. doi: 10.1007/s42380-019-00034-6

[ref23] Huppert F. A. Johnson D. M. (2010). A controlled trial of mindfulness training in schools: The importance of practice for an impact on well-being. J. Posit. Psychol. 5, 264–274. doi: 10.1080/17439761003794148

[ref24] Jalón C. Montero-Marin J. Modrego-Alarcón M. Gascón S. Navarro-Gil M. Barceló-Soler A.. (2020). Implementing a training program to promote mindful, empathic, and pro-environmental attitudes in the classroom: a controlled exploratory study with elementary school students. Curr. Psychol. 1–9. doi: 10.1007/s12144-020-00962-3 [Epub ahead of print]

[ref25] Johnstone J. M. Roake C. Sheikh I. Mole A. Nigg J. T. Oken B. (2016). School-based mindfulness intervention for stress reduction in adolescents: design and methodology of an open-label, parallel group, randomized controlled trial. Contemp. Clin. Trials Commun. 4, 99–104. doi: 10.1016/j.conctc.2016.07.001, PMID: 27754492 PMC5052085

[ref26] Kuyken W. Weare K. Ukoumunne O. C. Vicary R. Motton N. Burnett R.. (2013). Effectiveness of the mindfulness in schools Programme: non-randomised controlled feasibility study. Br. J. Psychiatry 203, 126–131. doi: 10.1192/bjp.bp.113.126649, PMID: 23787061

[ref27] López-Martínez M. (2004). “Principios y argumentos de la noviolencia,” in Manual de paz y conflictos. eds. Molina-Rueda B. Muñoz-Francisco A. (Madrid: EUG), 303–330.

[ref28] Loy D. (2018). Ecodharma: Buddhist Teachings for the Ecologycal Crisis. New York, NY: Wisdom Publications.

[ref29] Macbeth A. Gumley A. (2012). Exploring compassion: A meta-analysis of the association between self-compassion and psychopathology. Clin. Psychol. Rev. 32, 545–552. doi: 10.1016/j.cpr.2012.06.003, PMID: 22796446

[ref30] McKeering P. Hwang Y. S. (2019). A systematic review of mindfulness-based school interventions with early adolescents. Mindfulness 10, 593–610. doi: 10.1007/s12671-018-0998-9

[ref31] Mercanlioglu Ç. (2010). The relationship of time management to academic performance of master level students. Int. J. Bus. Manag. Stud. 2, 25–36.

[ref32] Modecki K. L. Minchin J. Harbaugh A. G. Guerra N. G. Runions K. C. (2014). Bullying prevalence across contexts: A meta-analysis measuring cyber and traditional bullying. J. Adolesc. Health 55, 602–611. doi: 10.1016/j.jadohealth.2014.06.007, PMID: 25168105

[ref33] Morris S. B. (2008). Estimating effect sizes from pretest-posttest-control group designs. Organ. Res. Methods 11, 364–386. doi: 10.1177/1094428106291059

[ref34] Nagler M. N. (2004). The Search for a Nonviolent Future. Makawao, HI: Inner Ocean Publishing.

[ref35] Nagler M. N. (2014). Nonviolence Handbook. New York, NY: Berrett-Koehler Publishers.

[ref36] Neff K. D. (2003). The development and validation of a scale to measure self-compassion. Self Identity 2, 223–250. doi: 10.1080/15298860309027

[ref38] Nelson-Gray R. O. Keane S. P. Hurst R. M. Mitchell J. T. Warburton J. B. Chok J. T.. (2006). A modified DBT skills training program for oppositional defiant adolescents: promising preliminary findings. Behav. Res. Ther. 44, 1811–1820. doi: 10.1016/j.brat.2006.01.004, PMID: 16579964

[ref39] Observatorio de la Convivencia Escolar (2016). Informe anual sobre el acoso escolar en la Comunidad de Madrid: curso 2015–2016. Madrid: MECD.

[ref40] Olweus D. (1996). Revised olweus bully/victim questionnaire: Evaluation in visually impaired. Optom. Vis. Sci. 90, 828–835. doi: 10.1097/OPX.0b013e3182959b52, PMID: 23792363

[ref41] Olweus D. Limber S. P. (2010). Bullying in school: evaluation and dissemination of the olweus bullying prevention program. Am. J. Orthopsychiatry 80, 124–134. doi: 10.1111/j.1939-0025.2010.01015.x, PMID: 20397997

[ref42] Olweus D. Solberg M. E. Breivik K. (2020). Long-term school-level effects of the Olweus bullying prevention program (OBPP). Scand. J. Psychol. 61, 108–116. doi: 10.1111/sjop.12486, PMID: 30277582

[ref43] Pullmer R. Chung J. Samson L. Balanji S. Zaitsoff S. (2019). A systematic review of the relation between self-compassion and depressive symptoms in adolescents. J. Adolesc. 74, 210–220. doi: 10.1016/j.adolescence.2019.06.006, PMID: 31254780

[ref44] Quach D. Jastrowski Mano K. E. Alexander K. (2016). A randomized controlled trial examining the effect of mindfulness meditation on working memory capacity in adolescents. J. Adolesc. Health 58, 489–496. doi: 10.1016/j.jadohealth.2015.09.024, PMID: 26576819

[ref45] Raes F. Pommier E. Neff K. D. Van Gucht D. (2011). Construction and factorial validation of a short form of the self-compassion scale. Clin. Psychol. Psychother. 18, 250–255. doi: 10.1002/cpp.702, PMID: 21584907

[ref46] Rawlett K. E. Friedmann E. Thomas S. A. (2019). Mindfulness based intervention with an attentional comparison group in at risk young adolescents: a pilot randomized controlled trial. Int. Med. Res. 8, 101–106. doi: 10.1016/j.imr.2019.04.002, PMID: 31193363 PMC6527909

[ref47] Salmoirago-Blotcher E. Druker S. Meleo-Meyer F. Frisard C. Crawford S. Pbert L. (2019). Beneficial effects of school-based mindfulness training On impulsivity in healthy adolescents: results From a pilot randomized controlled trial. Explore 15, 160–164. doi: 10.1016/j.explore.2018.07.003, PMID: 30309789 PMC9678328

[ref48] Santorelli S. (2000). Heal Thy Self. New York, NY: Crown Pulications.

[ref49] Schonert-Reichl K. A. Lawlor M. S. (2010). The effects of a mindfulness-based education program on pre- and early adolescents’ well-being and social and emotional competence. Mindfulness 1, 137–151. doi: 10.1007/s12671-010-0011-8

[ref50] Schwarz A. Schwarz A. (2017). Relajación Muscular Progresiva de Jacobson. Madrid: Editorial Hispano Europea.

[ref51] Singh S. (2016). Effects of mindfulness therapy in managing aggression and conduct problem of adolescents with ADHD symptoms. Indian J. Health Wellbeing 7, 483–487.

[ref52] Smith P. K. (2016). Bullying: definition, types, causes, consequences and intervention. Soc. Personal. Psychol. Compass 10, 519–532. doi: 10.1111/spc3.12266

[ref53] Suarez A. Lee D. Y. Rowe C. Gomez A. A. Murowchick E. Linn P. L. (2014). Freedom Project: Nonviolent communication and mindfulness training in prison. Sage Open 4:215824401351615. doi: 10.1177/2158244013516154

[ref54] Tharaldsen K. (2012). Mindful coping for adolescents: beneficial or confusing. Adv. Sch. Ment. Health Promot. 5, 105–124. doi: 10.1080/1754730X.2012.691814

[ref55] Ttofi M. M. Farrington D. P. (2011). Effectiveness of school-based programs to reduce bullying: A systematic and meta-analytic review. J. Exp. Criminol. 7, 27–56. doi: 10.1007/s11292-010-9109-1

[ref56] Turanzas J. (2013). Adaptación Transcultural de la Escala CAMM (Child and Adolescent Mindfulness Measure) y estudio preliminar de sus características psicométricas. master’s thesis. Valencian International University.

[ref57] Villanueva Badenes L. Usó Guiral I. Adrián Serrano J. (2013). Los programas de mediación entre iguales: una herramienta eficaz para la convivencia escolar. Apunt. Psicol. 31, 165–171.

[ref58] Wang H. (2018). Nonviolence as teacher education: a qualitative study in challenges and possibilities. J. Peace Educ. 15, 216–237. doi: 10.1080/17400201.2018.1458294

[ref59] Wilson A. C. Mackintosh K. Power K. Chan S. W. Y. (2019). Effectiveness of self-compassion related therapies: a systematic review and meta-analysis. Mindfulness 10, 979–995. doi: 10.1007/s12671-018-1037-6

[ref60] Wongtongkam N. Day A. Ward P. R. Winefield A. H. (2015). The influence of mindfulness meditation on angry emotions and violent behavior on Thai technical college students. Eur. J. Int. Med. 7, 124–130. doi: 10.1016/j.eujim.2014.10.007

[ref61] Yeager D. S. Fong C. J. Lee H. Y. Espelage D. L. (2015). Declines in efficacy of anti-bullying programs among older adolescents: theory and a three-level meta-analysis. J. Appl. Dev. Psychol. 37, 36–51. doi: 10.1016/j.appdev.2014.11.005

[ref62] Zenner C. Herrnleben-Kurz S. Walach H. (2014). Mindfulness-based interventions in schools-A systematic review and meta-analysis. Front. Psychol. 5:603. doi: 10.3389/fpsyg.2014.00603, PMID: 25071620 PMC4075476

[ref63] Zoogman S. Goldberg S. B. Hoyt W. T. Miller L. (2015). Mindfulness interventions with youth: A meta-analysis. Mindfulness 6, 290–302. doi: 10.1007/s12671-013-0260-4

