# Supplementary material for: Testing the Efficacy of ‘Unlearning’, a Mindfulness and Compassion-Based Programme for Cultivating Nonviolence in Teenagers: A Randomised Controlled Trial
Source: Front Psychol. 2021 Dec 16;12:717736. doi: 10.3389/fpsyg.2021.717736 (PMC8716809; doi:10.3389/fpsyg.2021.717736)
Supplement: Supplementary file 1 [file Table_1.docx]

**Supplementary table.** Bivariate correlations between the study variables.

|  | *ATSAS Cognitive* | *ATSAS Affective* | *ATSAS Behaviour* | *CAMM* | *SCS* |
| --- | --- | --- | --- | --- | --- |
| *ATSAS Cognitive* | - | **-.619**  **(< .001)** | **.670**  **(< .001)** | -.003  (.970) | **-.310**  **(< .001)** |
| *ATSAS Affective* | **-.619**  **(< .001)** | - | **-.590**  **(< .001)** | .021  (.790) | **.212**  **(.006)** |
| *ATSAS Behaviour* | **.670**  **(< .001)** | **-.590**  **(< .001)** | - | .059  (.456) | **-.301**  **(< .001)** |
| *CAMM* | -.003  (.970) | .021  (.790) | .059  (.456) | - | -.090  (.254) |
| *SCS* | **-.310**  **(< .001)** | **.212**  **(.006)** | **-.301**  **(< .001)** | -.090  .254 | - |

***Note***: p value can be found between brackets. In bold, statistically significant correlations.
